# Books Review

**Published:** 1870-12

**Authors:** 


### Books Review.

*Transactions of the American Medical Association.* 1870.

We are most happy to announce the early issue of this volume, which appears in its usual excellent style, but with unusual typographical accuracy. If we may receive the transactions of the society thus early, and in the unexceptionable manner that it is furnished, all propositions to change the style of publication will hereafter be very unanimously rejected: indeed, the idea of change is entirely inconsistent with the condition of things, and, as we believe, with the best interests of the association.

Members of the Association can receive the volume by forwarding their annual dues to the Secretary. We publish the table of contents for the benefit of those who are interested to know what the payment of their annual dues will secure to them.

We shall take occasion, hereafter, to speak more in detail of some of the papers presented; for the present we can only say, that the volume is of unusual interest, and that the secretary, Dr. Atkinson, has placed the American medical profession under lasting obligations to the ability, faithfulness and fidelity which has secured to them this volume thus early, and in such unexceptionable manner.

CONTENTS:—Minutes of the Twenty-first Annual Meeting of the American Medical Association; Report of the Committee of Publication; Report of the Treasurer; Address of George Mendenhall, M. D., President of the Association; Report of the Librarian; Report of the Committee on Medical Literature; Report of the Committee on Nomenclature of Diseases; Report on the Proper Treatment of the Insane, by John Curwen, M. D., of Harrisburgh, Pa.; Report of the Delegate to the Association of Superintendents of Asylums for the Insane for 1869; Report of the Committee appointed to Memorialize Congress on the Cultivation of the Cinchona Tree in the United States; Report of the Section on Anatomy and Surgery; A Paper on Median Lithotomy, by James L. Little, M. D.; New Operation on Imperforate Anus, by Thomas M. Healey, M. D; Form of Neuralgia of the Jaw-Bones, hitherto undescribed, by S. D. Gross, M. D.; Partial Paralysis from Reflex Irritation, caused by Congenital Phimosis and Adherent Prepuce, by Lewis A. Sayre, M.D.; Liquid for the Preservation of Wet Anatomical Preparations, etc., by B. Titcomb, M. D.; Report of a Case of Congenital Occlusion of the Rima Glottidis, by Louis Elsberg, A. M., M. D.; New Method of Reducing Dislocations at the Shoulder-Joint, by Samual Logan, M. D.; A Contribution to Plastic Surgery, by Gurdon Buck, M. D.; Case of Formation of Bone in the Eye—Enucleation, by Chas. M. Carleton, M. D.; A new Mode of Amputation at the Ankle-Joint, by I. N. Quimby, M. D.; A new Method of Lithotrity, by E. M. Moore, M. D.; Report of the Section on Medical Jurisprudence, Hygiene and Physiology;

On the Cellular Structure of the Red Blood Corpuscle, by Joseph G. Richardson, M. D.; Report on the Doctrine of Force, Physical and Vital, by J. H. Waters, M. D.; Report of the Section on Climatology and Epidemics; Report on the Diseases of the State of Pennsylvania for the Years 1867–68 and 1868–69, by D. Francis Condie, M. D.; Report on Topography, Climatology and Epidemic Diseases of the State of Illinois, by R. C. Hamill, M. D.; Report of the Section on the Practice of Medicine and Obstetrics; Intra-uterine Injections and their Therapeutical Value, by J. Byrne, M. D.; The Physiological Laws of Human Increase, by Nathan Allen, M. D.; Report on the Propriety of Establishing a Cinchona Plantation in the United States, by Thomas Antisell, M. D.; Report of the Section on Psychology; Report on American Necrology, by Christopher C. Cox, M. D., LL. D.; Prize Essay: An Essay on the Treatment of Aneurism, by Benjamin Howard, M. D.; Plan of Organization; Code of Medical Ethics; Catalogue of the Officers of the Association.

---

*The American Practitioner.* A Monthly Journal of Medicine and Surgery. Edited by DAVID YANDELL, M. D. and THEOPHILUS PARVIN, M. D., Louisville, Ky.

Through the favor of the Editors, we have received the first and second volumes of this Journal, elegantly bound in cloth. In again looking over this work, we are more forcibly than ever impressed with its excellence and worth. It shows that great labor has been bestowed upon the Journal; that it has contained in its monthly issues such articles, from the best authors, upon such subjects as are of deepest interest to the profession. All the various topics of medical and surgical interest have been presented in most complete and unexceptionable manner; and these volumes, as offered to us, reflect the greatest credit, not only upon the editorial conduct of the Journal, but upon the long list of able and well-known contributors. We hope the Journal may long maintain its present high rank, adding, as it now does to the influence and usefulness of American periodical medical literature; and that we may be the fortunate recipient of many future volumes.

---

*Hand-Book of Medical Microscopy.* By JOSEPH G. RICHARDSON, M. D. Philadelphia: J. B. LIPPINCOTT & Co. 1871.

The author has most completely accomplished his object, in furnishing a Manual of Practical Miscroscopy, to meet the demands of those who, from any cause, have been prevented from acquiring or retaining a due familiarity with the instrument and its requisite manipulations; or those who require aid in examining the urine, sputum, blood, &c., with the microscope. The author estimates that at least one-half of the cases of disease which physicians are called to treat, would have some light thrown upon their nature by a careful examination of the urine, blood, sputum, &c., with the microscope; and has endeavored, in his manual, to assist and promote the habit of making such examinations among medical men, believing that an earnest, conscientious physician can scarcely discharge his whole obligation to his patients without frequent resort to such examination. The excellence of the work is apparent in every page, and too much cannot be said in its favor.

The author has introduced into the work some new observations upon albuminuria, detection of blood stains, the identity of salivary, pus, and white blood corpuscles, and on the recognition of lung tissue as an aid to early diagnosis in consumption.

This little work appears eminently well adapted to the wants of the busy members of the profession, who can hardly find time to extract from the more voluminous works the instructions they require. If Prof. Beale and other authors have written more extensively, perhaps not more practically, and we really believe that the equal utility of the less expensive manual will be recognized.

